# Successful Management of Active Tuberculosis During Allogeneic Hematopoietic Stem Cell Transplantation in a Patient With Relapsed Acute Myeloid Leukemia: A Case Report and Literature Review

**DOI:** 10.7759/cureus.82026

**Published:** 2025-04-10

**Authors:** Maryame Ahnach, Zakaria EL Kodmiri, Inasse Mourabiti, Mounia Bendari, Bouchra Ghazi

**Affiliations:** 1 Immunopathology, Immunotherapy, and Immunomonitoring Laboratory, Faculty of Medicine, Mohammed VI University of Health and Sciences (UM6SS), Casablanca, MAR; 2 Department of Hematology, Cheikh Khalifa International University Hospital, Casablanca, MAR; 3 Department of Hematology, Faculty of Medicine, Mohammed VI University of Health and Sciences (UM6SS), Casablanca, MAR; 4 Department of Gynecology and Obstetrics, Mohammed VI International University Hospital, Casablanca, MAR

**Keywords:** acute myeloid leukemia (aml), hematopoietic stem cell, hsc transplantation, treatment, tuberculosis (tb)

## Abstract

Acute myeloid leukemia (AML) is a hematologic malignancy that often requires hematopoietic stem cell (HSC) allografting after relapse. Tuberculosis (TB) remains a significant concern in regions where it is endemic, posing a challenge in the management of these patients. A 38-year-old male with AML, who achieved complete remission after induction chemotherapy and three consolidation courses, relapsed one year later with additional chromosomal abnormalities. He received FLAG-Ida salvage chemotherapy and achieved both hematological and cytogenetic remission. During the pre-allograft check-up, an abdominal ultrasound revealed mesenteric adenopathies, and biopsy confirmed tuberculous adenitis. Given the urgency of HSC transplantation, the patient initiated anti-bacillary therapy (ERIP K4 capsules per day for three weeks) before starting his FB4 conditioning regimen. The therapy was continued during the transplant process. The patient completed six months of anti-bacillary treatment, with no TB reactivation observed at the latest follow-up. This case highlights the critical need for screening both donors and recipients for latent and active TB infection in endemic regions. Current literature supports the importance of pre-transplant TB screening and tailored management to address the complexities of TB treatment in stem cell transplantation, particularly in TB-endemic areas.

## Introduction

Tuberculosis (TB) is a significant challenge for immunocompromised patients, particularly those undergoing allogeneic hematopoietic stem cell transplantation (HSCT), in regions with moderate to high prevalence. In 2021, there were 10.6 million new TB cases globally and 1.6 million TB-related deaths, with Morocco reporting an incidence rate of 94 per 100,000 people [1]. The WHO highlights that the TB burden is higher in low- and middle-income countries, where healthcare disparities and socioeconomic factors play a major role. Morocco, with an estimated 35,000 new TB cases in 2021, underscores the need for strong TB control measures, especially for immunocompromised populations, including those undergoing allogeneic HSCT [1].

TB is a chronic infectious disease caused by *Mycobacterium tuberculosis*, primarily affecting the lungs but potentially involving other organs, especially in immunocompromised individuals. Acute myeloid leukemia (AML) is a malignant disorder of hematopoietic stem cells (HSCs) characterized by the rapid proliferation of immature myeloid cells, leading to bone marrow failure [1].

Managing TB in patients with hematologic malignancies undergoing HSCT is particularly challenging. The immunosuppressive treatments used to manage both the malignancy and the conditioning regimen before the transplant weaken the immune system. This makes patients more vulnerable to TB reactivation or progression. Normally, the immune system controls latent TB infection (LTBI), but the suppression of immune function in these patients increases the risk of reactivation. Additionally, the use of immunosuppressive drugs complicates the management of active TB infection (ATBI), making it harder to treat and leading to potentially more severe disease. The situation is further complicated by drug interactions between antitubercular medications and the medications used during the transplant process, which may affect the efficacy and safety of both treatments [2].

Patients with hematologic malignancies, particularly AML, are highly vulnerable to TB due to intrinsic immunosuppression and chemotherapy-induced cytotoxicity. HSCT further increases this risk with myeloablative regimens and prolonged post-transplant immunosuppressive therapy, creating significant challenges in managing TB while preventing transplant-related complications. Despite these risks, TB management in HSCT patients remains inadequately addressed in guidelines, especially in TB-endemic countries. Current screening does not account for the severe immunosuppression in transplant recipients, and there is a lack of standardized protocols for managing both TB and transplant therapies, including drug interactions and treatment initiation timing [3].

TB is particularly dangerous in HSCT patients because their immune systems are severely weakened by both their underlying disease and intensive treatments like chemotherapy and conditioning regimens. These therapies reduce the function of T-cells and other immune defenses, making it easier for LTBI to reactivate or for new infections to take hold. This increased vulnerability makes TB harder to control and more likely to lead to serious complications in this population [3].

Our observation in this case study reported TB during allogeneic HSCT, which faced many challenges. Moreover, these findings highlight the critical need for systematic TB screening in patients undergoing iatrogenic immunosuppression, particularly in regions with moderate to high TB prevalence. Notably, there is currently a lack of standardized guidelines addressing TB management in the context of HSCT in resource-limited countries such as Morocco. This case helps improve understanding of how to manage TB in HSCT patients by showing that ATBI can be treated during a transplant without harming its success. It provides real-world evidence that anti-TB treatment can be safely included in transplant care when closely monitored and tailored to the patient. This is especially important in countries with high TB rates, where clear guidelines are needed to manage drug interactions and weakened immune systems in these patients. Existing guidelines focus on LTBI screening but lack clear recommendations for managing ATBI during HSCT, particularly regarding drug interactions and immunosuppressive adjustments. This case report addresses critical gaps in current literature regarding TB management during HSCT, particularly in endemic regions, and provides insights for developing context-specific guidelines, especially regarding drug interactions and treatment adjustments.

## Case presentation

A 38-year-old patient with no significant pathological history or known TB exposure was diagnosed with AML (M4), according to the French-American-British classification system. The initial presentation was hyperleukocytic without disseminated intravascular coagulation or central nervous system involvement, and a favorable karyotype characterized by inv(16). Induction chemotherapy with the 3+7 regimen, daunorubicin (45 mg/m² for three days) and cytarabine (100 mg/m² for seven days), followed by three consolidation courses of high-dose cytarabine (3 g/m² twice daily on days 1, 3, and 5), achieved complete bone marrow remission.

**Table 1:** Summary of clinical and diagnostic findings in a relapsed AML patient AML: acute myeloid leukemia, HB: hemoglobin, PLT: platelet, WBC: white blood cells, CRP: C-reactive protein, PCT: procalcitonin, ECOG: Eastern Cooperative Oncology Group

Category	Observation
Presenting symptoms	Fatigue (asthenia), pallor, purpuric lesions, fever (38.5°C), productive cough lasting two months
Performance status	ECOG 1 (conscious, ambulatory)
Physical exam	No lymphadenopathy or splenomegaly; signs of anemia and infection
Key lab results	Pancytopenia (HB: 11.5 g/dL; PLT: 18 ×10³/μL; WBC: 5.5 ×10³/μL; neutrophils: 1.3 ×10³/μL)
	Elevated CRP (106 mg/L); PCT (0.1 ng/mL)
Bone marrow findings	65% blast infiltration; positive for myeloid blast markers on flow cytometry
Genetic results	Abnormal karyotype: 48, XY, +8, inv(16), +21; no mutations in IDH, NPM1, and FLT3
Differential diagnosis	Infection-related cytopenia, relapse of AML, other marrow failure syndromes
Final diagnosis	Relapsed AML

Given the relapse of AML with additional chromosomal abnormalities, the patient received salvage chemotherapy using the FLAG-IDA protocol, followed by intensification with allogeneic HSCT. The patient was prepared with infection management, transfusional support, and central venous catheter placement before initiating the FLAG-IDA regimen: fludarabine (50 mg/m²) and cytarabine (2 g/m²) for five days, with idarubicin (10 mg/m²) added on days 3 and 4. The post-chemotherapy course was complicated by severe aplasia, infection, and staphylococcal sepsis, requiring management with broad-spectrum antibiotics (imipenem/cilastatin, vancomycin) and antifungal therapy (voriconazole), along with transfusion support. Post-treatment evaluation showed a normal hematological count (hemoglobin: 12.2 g/dL, platelet: 223,000/μL) with no blasts in the bone marrow and a normal karyotype, concluding complete hematologic and cytogenetic remission. Human leukocyte antigen typing identified compatibility with the patient’s 30-year-old sister, making the patient eligible for a genotypically identical allogeneic HSCT.

**Figure 1:**
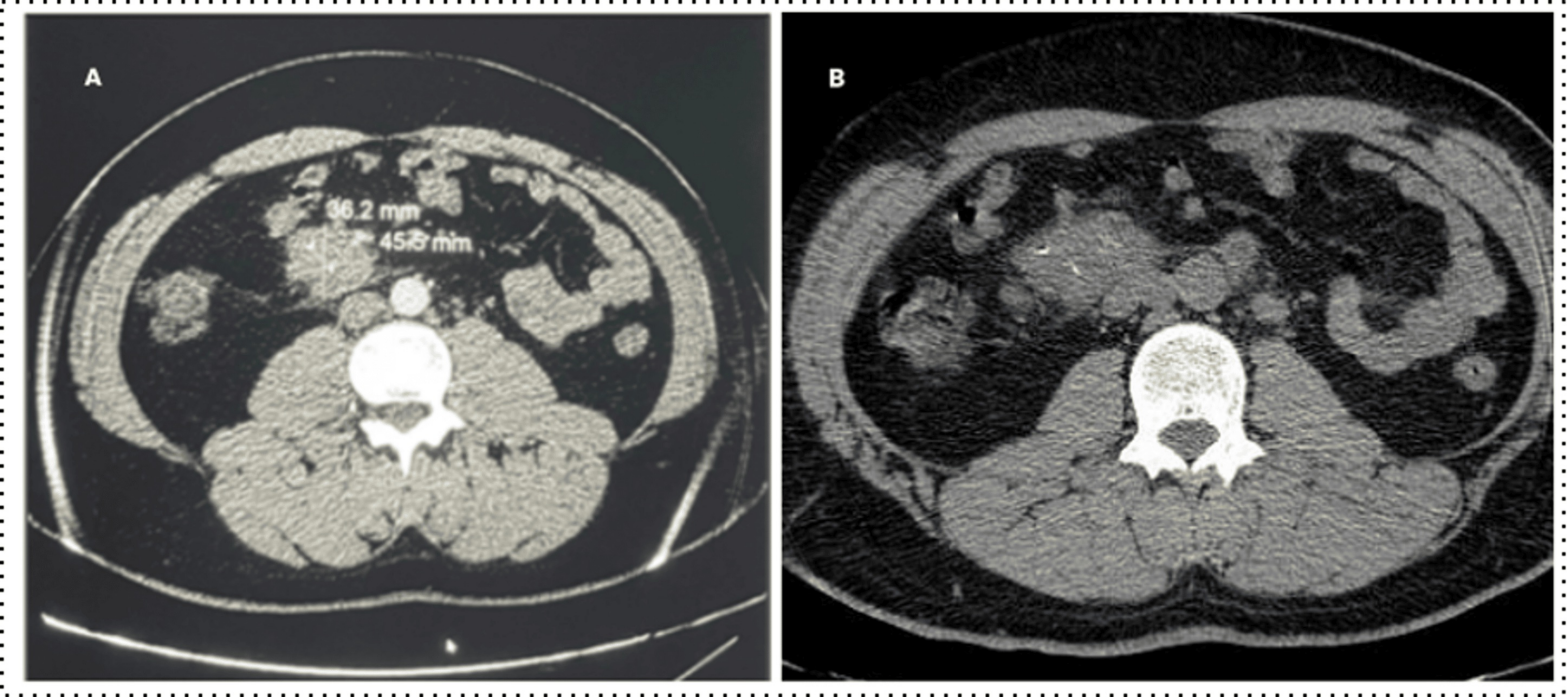
CT images of the patient demonstrating abdominal lymphadenopathy (A) Axial section of a contrast-enhanced abdominal-pelvic CT scan showing a round celiac-mesenteric lymphadenopathy, measuring 36.2 mm and 45.5 mm in diameter with enhancement. (B) Axial section of a non-contrast-enhanced CT scan showing a mesenteric lymphadenopathy with calcifications typical of TB. CT: computed tomography, TB: tuberculosis

During the pre-transplant assessment, renal and liver function were normal. The infectious screening was negative (cytomegalovirus, Epstein-Barr virus, herpes, syphilis, toxoplasmosis, aspergillosis). Radiological evaluation revealed centimeter-sized mesenteric lymphadenopathy on the abdominal CT scan (Figure 1). The polymerase chain reaction and GeneXpert tests on blood samples were negative. A biopsy was performed, showing a morphological aspect of evolving caseo-follicular tuberculous adenitis. The patient experienced a weight loss of 3 kg over the course of six days.

Given the risk of leukemia relapse and the urgency of HSCT, the patient was initiated on anti-TB therapy with ERIP K4 (isoniazid (INH), rifampicin, pyrazinamide, and ethambutol), four capsules per day for three weeks prior to starting the FB4 conditioning regimen. Anti-TB treatment was continued throughout the allograft process without interruption.

HSCs were collected in peripheral blood cells with adequate graft richness confirmed (CD34/kg: 10 6). The post-transplant aplasia period lasted 13 days and was complicated by grade 4 mucositis and staphylococcal infection with sepsis, both managed successfully with appropriate antibiotic therapy. Importantly, no complications related to TB, hepatic toxicity, or renal toxicity were observed during this period.

Chimerism analysis on day 30 post-transplant demonstrated 97.47% donor DNA contribution. The patient completed the six-month anti-TB treatment course without any signs of TB reactivation or graft-versus-host disease (GVHD) reported to date. The patient completed his six months of anti-bacillary treatment, and no symptoms of TB reactivation or signs of GVHD have been reported to date.

## Discussion

The management of TB in HSCT recipients presents unique challenges that require careful consideration of timing, drug interactions, and monitoring protocols. The spectrum of TB manifestation in HSCT candidates ranges from LTBI to ATBI, with varying clinical presentations. While pulmonary TB remains most common, extra-pulmonary manifestations like our patient's mesenteric lymphadenitis require particular attention during pre-transplant screening [4].

TB screening tools

The limitations of current diagnostic tools in immunocompromised patients warrant consideration. The tuberculin skin test (TST) and interferon-gamma release assays (IGRAs) show reduced sensitivity in immunocompromised patients, potentially leading to false-negative results. Molecular methods like GeneXpert MTB/RIF, while rapid and specific, may not detect extra-pulmonary TB effectively, as evidenced by our patient's negative PCR results despite histologically confirmed disease [4,5]. This underscores the importance of maintaining high clinical suspicion and utilizing multiple diagnostic modalities in pre-transplant evaluation.

TB can present in two basic forms: ATBI and LTBI. ATBI, which is symptomatic, presents clinically as fever, weight loss, chronic cough, and organ-specific symptoms in more severe cases, depending on the localization of TB. Diagnosis may be confirmed microbiologically by tests including acid-fast bacilli staining, nucleic acid amplification tests, and culture or pathologically via histological examination of affected tissues [6]. In contrast, LTBI is asymptomatic and is characterized by the immune response successfully controlling *Mycobacterium tuberculosis* without clinical disease. Nevertheless, with LTBI, there is the risk of reactivation, especially in conditions of immunosuppression, such as allogeneic HSCT. Diagnosis depends on screening tools such as the TST and IGRAs. They are used to assess immune sensitization to TB antigens and ascertain candidates for preventive treatment. Chest radiography can also be applied as an add-on for the investigation of probable ATBI in suspected patients [7].

TB risk factors

Systematic risk assessment and monitoring protocols are essential for TB management in HSCT. Pre-transplant risk factors, including prior TB exposure, history of TB, type of conditioning regimen, donor type, and anticipated immunosuppression duration, must be thoroughly evaluated to determine the patient’s vulnerability to TB [8]. Risk factors for TB include a history of previous exposure to *Mycobacterium tuberculosis*, such as a positive purified protein derivative result or radiological evidence of untreated previous TB. Pretransplant clinical conditions contributing to TB risk include the recipient's age, chronic renal insufficiency or hemodialysis in kidney transplant patients, diabetes mellitus, hepatitis C virus in kidney transplant patients, chronic liver disease, and other coexisting infections like severe mycoses, cytomegalovirus, *Pneumocystis jirovecii*, or *Nocardia pneumonia*. Immunosuppressive therapy also increases the risk, including the use of OKT3 or anti-T-lymphocyte antibodies, intensification of immunosuppression associated with graft rejection, the combination of mycophenolate mofetil and tacrolimus versus azathioprine-cyclosporine-prednisone, and the use of everolimus in lung transplantation [8]. Post-transplant monitoring is essential, incorporating regular clinical assessments, periodic imaging, surveillance for drug-related complications, and evaluations of immune reconstitution to promptly detect TB reactivation and ensure the safety of the transplant regimen.

Literature review

Our case report represents the successful management of ATBI before allogeneic HSCT in a patient with relapsed AML. Similarly, we present another study of Liu et al., who studied seven leukemia patients with ATBI who received a triple or quadruple anti-TB regimen for three months prior to allogeneic HSCT, resulting in symptom improvement but incomplete TB resolution. During HSCT, intravenous moxifloxacin and amikacin replaced oral anti-TB drugs, and the original regimens resumed post-hematopoietic recovery. A double anti-TB regimen was used for six months post-transplant and discontinued after one year. One patient with AML-M7 required prolonged anti-TB therapy due to chronic GVHD. All patients achieved complete leukemia response pre-transplant, with bone marrow hyperplasia observed within 30 days post-transplant. During follow-up, three developed grade II acute GVHD, and two had extensive chronic GVHD, all resolving with immunosuppression. Most patients maintained complete remission, except two who relapsed but responded to further treatment. Lastly, no TB infection-related death was reported in his study [9].

TB Management and Treatment Recommendations in HSCT

Evaluation of HSCT candidates: Based on the American Society for Blood Marrow Transplantation guidelines, HSCT candidates should be screened for TB based on Centers for Disease Control and Prevention guidelines, including history of ATBI, exposure to high-risk contacts, and results of TST or IGRAs [7]. While IGRAs are more specific, a negative result does not exclude LTBI. Candidates with negative IGRA results have a low risk of developing ATBI post-transplant [7].

Management of positive TB screening: Candidates with positive TST or IGRA must undergo further evaluation, including a chest X-ray, to rule out ATBI. ATBI requires isolation and treatment before HSCT, while LTBI can be treated without delaying the transplant [10].

Evaluation of HSCT donors: The risk of TB transmission from donors is negligible, so routine screening for LTBI is not recommended. If ATBI is detected in a donor, HSCT should be delayed until treatment is completed and the donor is non-infectious [10].

Treatment of TB infection: For LTBI, the American Society for Blood Marrow Transplantation guidelines and the European Society of Clinical Microbiology and Infectious Diseases recommend INH 300 mg daily with pyridoxine (vitamin B6) for nine months as the primary treatment for LTBI [11]. INH has been proven effective in preventing TB. The therapy duration and dosage are consistent regardless of timing relative to transplantation, and patients who complete INH treatment before transplantation do not require it again. Monitoring for hepatotoxicity is essential, though INH is generally well tolerated, with limited interaction with calcineurin inhibitors [3]. Alternative prophylactic regimens include INH at a dose of 900 mg twice weekly for nine months via directly observed therapy (DOT), rifampin at 600 mg daily for four months (with or without INH), or a combination of weekly rifapentine with INH for three months using DOT [11]. These shorter regimens improve therapy completion rates before transplantation and are associated with fewer side effects. However, the combination of rifampin and pyrazinamide is no longer recommended due to its high risk of severe liver toxicity [12]. For HSCT transplant recipients with suspected ATBI, initial treatment should involve three drugs: INH, ethambutol, and pyrazinamide [13]. Careful consideration is needed when including rifampin or rifapentine in the treatment regimen due to their significant interactions with immunosuppressive medications. Treatment should ideally start before conditioning therapy or immediately after if necessary, based on the risk of disease progression [13].

For ATBI, the recommended initial regimen consists of a combination of INH, ethambutol, and pyrazinamide [13]. In severe cases, the inclusion of a fluoroquinolone is advised. After eight weeks, treatment can be continued with INH alone if cultures are confirmed to be negative [14].

TB Management Challenges and Limitations in HSCT

In TB-endemic areas, transplant centers must implement robust pre-transplant TB screening protocols and tailor center-specific guidelines based on local epidemiology. The timing of HSCT in relation to TB therapy remains a subject of debate, but initiating anti-tubercular therapy three weeks prior to conditioning allows for the reduction of mycobacterial load, ensures tolerability of treatment, and minimizes the risk of reactivation during transplantation [15]. Drug interactions, particularly with rifampicin-based regimens, necessitate careful monitoring due to potential interference with transplant medications. Treatment duration may also require extension beyond the standard six-month regimen, with individualized adjustments based on clinical response and immune reconstitution, ensuring optimal TB management and HSCT outcomes [15].

The management of TB in HSCT patients requires specialized isolation protocols and a multidisciplinary approach involving transplant teams, infectious disease specialists, and TB experts to ensure comprehensive care and patient safety [16]. Enhanced monitoring is crucial to detect complications, optimize therapeutic outcomes, and address key gaps, such as the optimal duration of pre-transplant TB treatment, the role of newer anti-tubercular agents, and the impact of conditioning regimens on TB reactivation. Systematic TB screening for allogeneic HSCT candidates, particularly in high-endemic regions, is vital to prevent LTBI activation. At the same time, regular monitoring of drug levels and interactions with immunosuppressive agents minimizes risks. Tailored, center-specific guidelines informed by local epidemiology, resources, and regional registries could standardize and enhance TB management [10]. However, limited data on treatment regimens, therapy duration, and drug interactions in transplant recipients underscores the need for further research and a collaborative approach to refining care strategies.

## Conclusions

In Morocco, TB remains endemic, and early detection and management of both LTBI and ATBI are crucial, especially for patients undergoing allogeneic HSCT. Comprehensive pre-transplant screening and ongoing monitoring during the transplant process are essential to prevent TB reactivation. Key risk factors for TB after transplantation include non-related donor stem cells, intensive conditioning therapies, and chronic graft-versus-host disease. Prophylactic INH therapy may be recommended, but gaps in guideline application and limited data on optimal management complicate treatment. Chest CT is important for diagnosing ATBI in HSCT patients because it can find early or unusual signs of the disease, like cavitary lesions or nodules, that may not show up on a regular chest X-ray. This is particularly useful for immunocompromised patients, where the usual symptoms of TB might not be clear. Chest CT helps confirm the diagnosis and helps doctors decide on the best treatment approach.

This case highlights the feasibility of managing TB and HSCT concurrently with appropriate monitoring. We recommend implementing systematic pre-transplant TB screening, developing standardized guidelines for anti-tubercular therapy timing, and creating region-specific management protocols. Further research is needed to optimize treatment strategies and improve outcomes in this complex patient population in TB endemic regions.
